# Development and Application of Aptamer-Based Surface-Enhanced Raman Spectroscopy Sensors in Quantitative Analysis and Biotherapy

**DOI:** 10.3390/s19173806

**Published:** 2019-09-03

**Authors:** Hai-Xia Wang, Yu-Wen Zhao, Zheng Li, Bo-Shi Liu, Di Zhang

**Affiliations:** College of Pharmaceutical Engineering of Traditional Chinese Medicine, Tianjin University of Traditional Chinese Medicine, Tianjin 301617, China

**Keywords:** Surface-enhanced Raman scattering, Raman scattering, aptamers, sensor, detection, hot spots, photothermal therapy

## Abstract

Surface-enhanced Raman scattering (SERS) is one of the most special and important Raman techniques. An apparent Raman signal can be observed when the target molecules are absorbed onto the surface of the SERS substrates, especially on the “hot spots” of the substrates. Early research focused on exploring the highly active SERS substrates and their detection applications in label-free SERS technology. However, it is a great challenge to use these label-free SERS sensors for detecting hydrophobic or non-polar molecules, especially in complex systems or at low concentrations. Therefore, antibodies, aptamers, and antimicrobial peptides have been used to effectively improve the target selectivity and meet the analysis requirements. Among these selective elements, aptamers are easy to use for synthesis and modifications, and their stability, affinity and specificity are extremely good; they have been successfully used in a variety of testing areas. The combination of SERS detection technology and aptamer recognition ability not only improved the selection accuracy of target molecules, but also improved the sensitivity of the analysis. Variations of aptamer-based SERS sensors have been developed and have achieved satisfactory results in the analysis of small molecules, pathogenic microorganism, mycotoxins, tumor marker and other functional molecules, as well as in successful photothermal therapy of tumors. Herein, we present the latest advances of the aptamer-based SERS sensors, as well as the assembling sensing platforms and the strategies for signal amplification. Furthermore, the existing problems and potential trends of the aptamer-based SERS sensors are discussed.

## 1. Introduction

**Figure 1:**
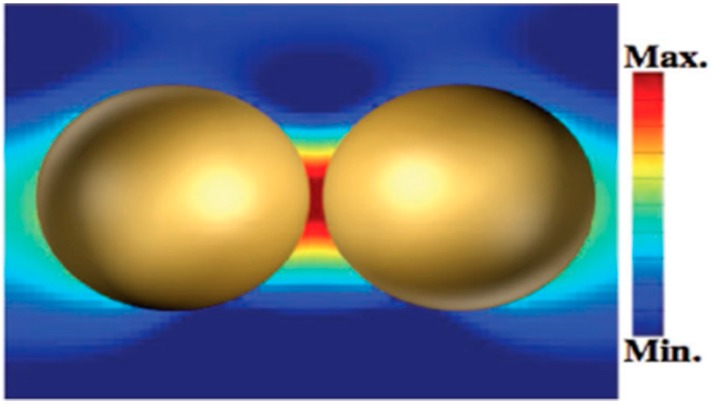
Representative electric field distribution of surface-enhanced Raman scattering (SERS)-active Au nanoparticles (NPs) at a “hotspot” site. (Reprinted with permission from Wiley, 2018 [11]).

As an emission technique, Raman spectroscopy is based on an inelastic scattering process involving an energy transfer between incident light and illuminated target molecules, which can be used for chemical identification, structure elucidation, and other qualitative analysis. However, the Raman signal is very weak because only approximately one in 10^8^ incident photons are Raman-shifted photons. That is why Raman spectroscopy has been discovered very early [1], but was not widely used until the appearance of surface-enhanced Raman scattering (SERS). The SERS effect was first discovered by Fleischman et al. [2] in 1974. They found a strong Raman scattering signal when a single layer of pyridine molecules was adsorbed on the surface of the rough silver electrode. Subsequently, the research groups of Jeanmaire and Creighton confirmed this experimental result and calculated the average Raman signal of each pyridine molecule in Fleischman’s experiment was enhanced by 10^6^ times, which was called the SERS effect [3,4]. Although the SERS enhancement mechanism is still not clear, two commonly recognized mechanisms are electromagnetic enhancement and chemical enhancement [5,6,7]. Among them, the electromagnetic enhancement mechanism makes a more significant contribution to the SERS [8]. In the electromagnetic theory, an obvious enhancement effect can be observed at the vicinity of the nanostructure surface, especially in the gap of the dimer of the Au, Ag nanoparticles (NPs) or the heterodimers themselves, which was called the “hotspots” effect [9,10,11] (Figure 1). Therefore, Au and Ag NPs were often used as SERS substrates and have been widely utilized in the detection of small molecules, biomacromolecules, and toxin molecules [12,13,14]. In chemical theory, a primary change in the polarizability of the molecule can be deduced by the charge transfer between the adsorbate molecule and the metal surface. Compared with the electromagnetic enhancement effect, the enhancement factors of chemical enhancement is about 10^3^, significantly lower than that of electromagnetic enhancement (EF of ~10^10^–10^11^) [15]. As a result, most SERS-based sensors in this review are developed by utilizing the electromagnetic enhancement effect.

Ray et al. developed a label-free SERS probe formed by several “hotspots” between the Au NPs and cysteine modified Au NPs to detect TNT with high selectivity and good sensitivity (with a 2 pM limit of detection—LOD) [16]. Besides, Zhou et al. designed a natural SERS substrate based on Au@Ag nanoparticle-coated mussel shell. The large-scale three-dimensional (3D) substrates exhibited an apparently enhanced Raman effect and were successfully used for the direct discrimination of *Staphylococcus aureus*, *Escherichia coli* and *Pseudomonas aeruginosa* [17]. Recently, Yin et al. prepared a hydrophobic paper-based SERS substrate by dispersing Ag NPs into commercial filter paper. The as-prepared SERS substrate platform could be directly used for the determination of melamine with high reproducibility and a low LOD value [18]. In addition, the novel hydrophobic SERS substrate could also be applied successfully in the determination of pesticides and dyes in the environment with high reproducibility in Raman intensity [19,20]. 

Generally speaking, the label-free SERS technology mentioned above presented a good prospect in detecting small hydrophilic or high polar molecules which have high affinity to SERS substrates, such as Au and Ag NPs. On the contrary, it is a great challenge to use them in detecting hydrophobic or non-polar molecules, especially in a mixed sample matrix or at low concentrations. For instance, it is difficult for discriminate among different kinds of bacteria from a mixed sample matrix. In addition, traditional Chinese medicine samples are another typical complex system. Multi-components in these samples could cause strong interference for target detection. Moreover, semi-micro and trace detection imposes strict requirements on the sensitivity of the detection method. In this context, increasing matrix selectivity may be a good solution to improving detection sensitivity. Antibodies [21,22], aptamers [23,24], antibiotics [25] and antimicrobial peptides [26] are common recognition elements with high specificity to the target molecules. Among them, aptamers are the most promising because of their low cost and good stability. As a group of single strand DNA or RNA, aptamers are screened by systematic evolution of ligands by exponential enrichment (SELEX) technology, and can be obtained by chemical synthesis. The end group of these oligonucleotides can be flexibly modified with different active groups, which provides the convenience to design and assemble of the aptamer-based biosensors in specific and practical application. Besides, the signal of SERS probes based on aptamer can be dramatically enhanced owing to the specific recognition between the aptamers and the targets [27,28]. Similarly, the apparently enhanced Raman effect can also be observed in the detection of small molecules, pathogenic microorganism, mycotoxins, tumor marker and other functional molecules by combination with aptamer. 

The aim of our review is to introduce the different types of SERS probes based on aptamers that have a strong Raman enhancement effect and high specific recognition ability. The promising substrates of SERS with different metallic nanosubstrates and their composite nanosubstrates are presented. Besides, the relationship between the Raman enhancing activity of the nanosubstrates and the size or the shape of these substrates is introduced. Moreover, the assembly of portable sensing platforms and the strategies in signal amplification based on the aptamers modified the SERS sensors are the key issue in the sensing area. As a result, this review describes them in detail along with their satisfying analysis results in determination and biotherapy applications. Finally, the challenges and outlook of the aptamer-based SERS sensors are discussed. In summary, our review summarizes the construction and application of aptamer conjugated SERS platforms systematically, which can be a good reference for the assembling and analytical application of aptamer-modified SERS sensors. 

## 2. Substrates of SERS

**Figure 2:**
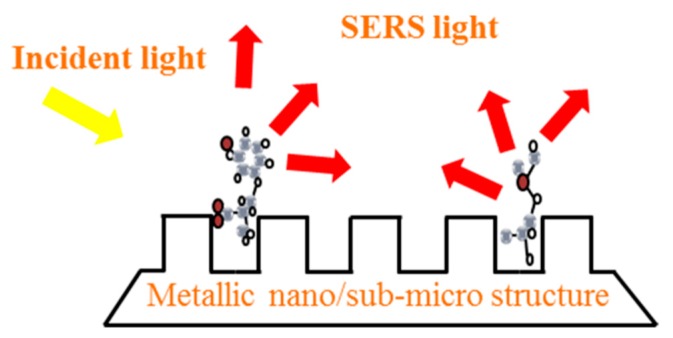
The schematic diagram of the enhancement of the Raman scattering light on the metallic nano/sub-microstructure surface.

The enhancement of Raman scattering light is closely related to the properties of the SERS substrates. The SERS substrates have metal selectivity and only a few metal materials can exhibit SERS effect, such as Au, Ag, Cu, basic lithium metal, and a part of the transitional metal [1]. Besides, the SERS substrates have structure selectivity, only nano or micro-scaled metal structures exhibit the SERS effect. In addition, the intensity of the SERS is closely related to the roughness of the material surface. As shown in Figure 2, when a metallic nano/sub-micro structure is exposed to the light, an interaction occurs between the light and the electrons of the metal surface, thus creating an enhanced electric field. This effect improves the Raman scattering light of the molecules that are close or attach onto the metal surface. There are about three kinds of SERS substrates, including rough metal electrodes, metal nanomaterial and nanocomposite structures.

### 2.1. Rough Metal Electrode

The SERS effect was first observed on the surface of a rough silver electrode in 1974. Nowadays, researchers have already understood that the SERS effect is closely related to the roughness of the electrode surface. Several methods have been used for improving the roughness of the electrode, including electrochemical redox, electrochemical step potential and cyclic voltammetry method [29,30,31]. Properly using these methods can achieve high Raman intensity [4]. However, the biggest drawback of these methods is poor reproducibility in the electrode preparation process, which makes the Raman effect unstable. Thus, these methods have rarely been used for enhancing the Raman scattering intensity.

### 2.2. Metal NPs in Suspension

Suspensions of metal NPs prepared by chemical methods are one of the most widely used SERS substrates. As they have several advantages, like easy preparation and handing, metal NPs’ size and shape can be easily controlled by tuning the reactant ratio and addition time [32,33]. Therefore, Au NPs, Ag NPs and their composite NPs are the most common metallic nanosubstrates for SERS. 

Lee and Meisel investigated the adsorption of the negatively charged dyes on the surface of Au NPs, Ag NPs and copper NPs and checked it by SERS technology. A highly efficient SERS signals of the dyes were observed on Au NPs and Ag NPs, while a less efficient SERS was shown for copper NPs [34]. Their research indicated that the Raman enhancing activity of the NPs was related to the type of metal. Besides, several studies indicated that the Raman enhancement effect was also related to the size of the metal NPs [35,36]. Christopher and Tuan synthesized types of high-yield Au nanostars ranging from 45 to 116 nm in size by seeding growth method [37]. *p*-Mercapto benzoic acid (*p*-MBA) was used as a Raman probe for the evaluation of the SERS activities of these Au nanostars in different sizes. The results showed that the SERS enhancement efficiencies were related to the size of nanostars, and the enhancement factor estimated as 5 × 10^3^ averaged over the 52 nm nanostars at 633 nm excitation. At the same time, the shape of the NPs is also an important factor on the Raman enhancement effect [38]. On the one hand, an increase in the sharpness of the edge of the NPs could increase the intensity of the surface plasmon resonance, which in turn enhances the SERS effect [15,38]. On the other hand, the NPs with multi-branched or sharp-edged structure could create more “hotspots”, which is very important for the enhancing of the Raman effect. Isabel et al. proved the correlation between the SERS intensity and the shape of the NPs [39] obtained via seeded growth in concentrated solutions. The research of Esenturk and Walker indicated that the SERS activity of the nanostars was notably stronger than that of the spherical or rod-shaped Au NPs of similar size [40]. Xu et al. developed an Au nanoflowers as SERS-active probes. A highly sensitive detection can be obtained by this novel structure of NPs [41]. 

### 2.3. Nano-composite Structures

Nano-composites formed by metal alloy are common SERS substrates. Their fabrication methods have been investigated and their properties have been sought for sensing applications. The groups of Yang and Guo prepared a type of heterostructured cubic Au-Ag composite, which was used as an effectively active SERS substrate in rapid detection of antibiotic ciprofloxacin [42]. Zhang et al. fabricated core-shell Au-Pt co-nanoparticle film, and this kind of film exhibited highly efficient catalytic properties and SERS activity, which could potentially be used in Raman sensing applications [43]. Besides these alloy nanocomposites, graphene oxide (GO), carbon nanotube (CNT) and magnetic nanoparticles (MNPs) are also commonly used as SERS substrates for enhancing the Raman signal. Blinking effects occurred frequently when the metallic materials were excited in the Raman detection process, and the generated high thermal radiation could cause the temporal intensity fluctuations. GO was used to avoid or reduce this effect, and the results showed that the thermal radiation could be reduced by approximately 79% in the GO/Au NPs nanocomposites [44]. Besides, Zhou et al. [45] prepared CNTs@SiO_2_@Ag nanocomposites by loading Ag NPs on the surface of multi-walled CNTs with SiO_2_ as the intermediate layer. The results showed that the intensity of Raman signal of Rhodamine 6G molecule was significantly enhanced. Therefore, the prepared CNTs@SiO_2_@Ag nanocomposite could be potentially used as a SERS substrate in the field of biological non-destructive testing. Additionally, magnetic NPs are always used as SERS substrates for enhancing the SERS intensity or improving detection sensitivity. Xiang et al. [46] described a new SERS substrate by conjugating Fe_3_O_4_ magnetic NPs with Au-Ag nanocomposites. Cytidine could be captured in the vicinity of the SERS hotspots under the external magnetic field, an evident Raman signal was detected at 784 cm^−1^ and the cytidine was quantified as low as 1 nM.

## 3. Aptamer-Based SERS Probes in Quantitative Analysis and Biotherapy

Due to the specific recognition between the aptamers and the targets, the aptamer-based SERS sensors can effectively improve the matrix selectivity and the detection sensitivity. The matrix selectivity can be evaluated by measuring the interaction between the aptamer and the target, while the detection sensitivity is determined by the intensity of the Raman signal. There are two types of aptamer-based SERS sensors: Those with and without the Raman signal molecule. The latter case is also commonly referred to as label-free mediated SERS approach. In this label-free SERS approach, some specific biological structures can be formed and the Raman signal can be directly determined [47]. However, most molecular species that could not give rise to their own Raman signal so that their Raman signal cannot be determined directly. Therefore, Raman signal tags or labels are usually utilized on/in to the molecular species and the detection of the target molecule is performed indirectly. In this review, most of the quantitative analyses are performed indirectly. Various aptamer-based SERS sensors with the Raman signal molecule were successfully used in the analysis of small molecules, pathogenic microorganism, mycotoxins, tumor marker and other functional molecules, as well as in biotherapy.

### 3.1. Determination of Small Molecules and Ions 

With the widespread use of pesticides and fertilizers, some toxic small molecules and heavy metal ions such as bis(2-ethylhexyl) phthalate (DEHP), nitrite, and Hg^2+^ are unavoidable. Several studies have demonstrated those molecules and ions are serious threats to human health and the environment [48,49,50]. Sensitive detections for these toxic substances have been achieved by using gas chromatography-mass spectrometry (GC/MS), inductively coupled plasma mass spectrometer, and high performance liquid chromatography (HPLC) with UV and diode array detectors [51,52,53,54,55]. However, the expensive and bulky instruments, complex sample operational procedures and professionally trained operators greatly limit the widespread using of such methods. Besides, enzyme linked immunosorbent assay (ELISA) immunoassays, sensors based on electrochemistry and fluorescence have been developed and achieved satisfying analysis results in the rapid detection of these substances [56,57]. The costly antibodies and low-sensitivity sensors are becoming new problems for the detection process. Therefore, it is urgent to develop simple, sensitive and easy-to-operate methods for the quantitative detection of toxic small molecules and ions. 

Nowadays, specific recognition aptamers for some ions and molecules have been developed and are gaining increasing attention because of their low cost, good stability and biocompatibility [58,59]. As identification probes, aptamers were widely used in quantitative analysis of small molecules and ions by combination with UV, fluorescence and electrochemistry technology. However, few studies have been conducted by the SERS technique combined with aptamer probes in this area.

Tu et al. designed a competitive binding assay for DEHP determination by aptamer modified MNPs and SERS silica particles [60]. On the one hand, the MNPs were functionalized with the DEHP aptamers by streptavidin–biotin interaction. On the other hand, the DEHP molecules were immobilized on the silica particles with (5, 5’-Dithiobis-(2-nitrobenzoic acid)) (DTNB) as a SERS reporter probe. In the presence of DEHP samples, the free DEHP molecules competed with the DEHP modified SERS silica particles to bind with the aptamer on the MNPs. After magnetic separation, the SERS signal of the free SERS silica particles could be detected, while the signal was positively correlated with the DEHP concentration. The trace DEHP could be detected by aptasensor and a portable Raman spectrometer with a LOD of 8 pM. Importantly, the aptasensor showed high selectivity and short detection time, which can be directly used in food and environment sample determinations.

**Figure 3:**
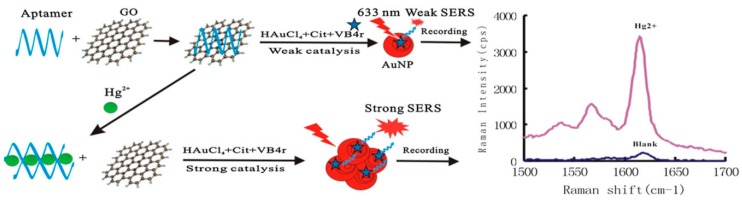
Scheme of aptamer-regulated graphene oxide (GO) catalysis and SERS detection of Hg^2+^ using VB4r as a molecular probe. (Reprinted with permission from Wiley, 2018 [61]).

Jiang’s group developed a sensitive SERS method for trace Hg^2+^ determination based on the co-regulated reduction of HAuCl_4_ by aptamer and GO. As shown in Figure 3, the positively charged aptamer can be easily adsorbed onto the GO surfaces owing to the electrostatic interaction. A significant inhibitory catalysis effect of GO for HAuCl_4_ reduction could be recorded at 1615 cm^−1^ by using Victoria blue 4R (VB4r) as a Raman probe. In the presence of Hg^2+^ the aptamer for Hg^2+^ could separate the aptamer from the GO surface by forming stable Hg^2+^-aptamer complexes. As a result, an obvious GO catalysis effect was recovered and an increased SERS signal was proportional to the concentration of Hg^2+^. The proposed platform by combination of specific recognition of aptamers and sensitive effect of SERS can be used to detect Hg^2+^ with a LOD of 0.08 nM [61].

**Figure 4:**
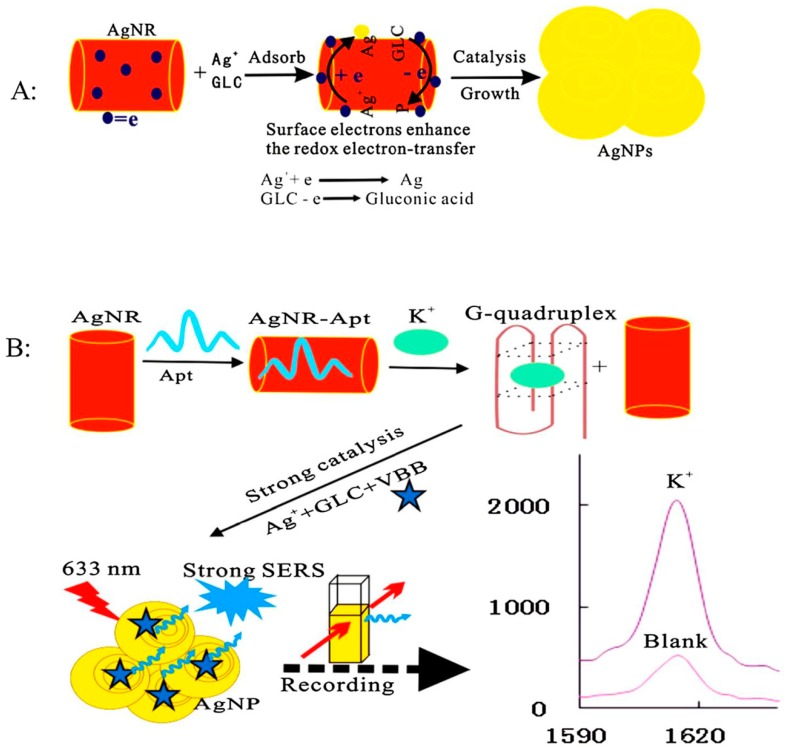
The principle of Ag nanoplasmon SERS detection of trace potassium by aptamer regulating AgNR catalysis. (**A**): Schematic diagram of the production process of AgNPs; (**B**): Schematic diagram of the detection process of K^+^. (Reprinted with permission from Elsevier, 2019 [62]).

Besides, the aptasensor based on SERS technique can also be used in the detection of essential ions of human bodies. The groups of Liang and Jiang designed a nanoAg platform for trace potassium detection by using SERS technique [62]. The detection principle was similar to the above mentioned for Hg^2+^ detection. The K^+^ aptamers could be analogously adsorbed onto the Ag nanorods (AgNR) surfaces based on the electrostatic interaction. Then, the AgNR catalysis effect on the redox reaction between glucose (GLC) and AgNO_3_ was decreased, while the SERS signal at 1615 cm^−1^ with Victoria blue B (VBB) as signal probes was weak. In the presence of K^+^, the situation was totally different: The formed stable G-quadruplex of K^+^-aptamer made the aptamer escape from the surface of AgNR, and the catalysis effect of AgNR was recovered. A strong catalysis effect can be recorded by a SERS technique with the increasing of the K^+^ concentration. A good linear relationship between the changed intensity of SERS signal and the concentration of K^+^ could be established in the range of 50–3000 nmol/L with an LOD of 25 nM (Figure 4).

### 3.2. Determination of Pathogenic Microorganism

Rapid detection of pathogenic microorganisms has always been a serious topic for human health. Conventional colony forming units counting-based methods are quite time-consuming. Molecular biological detection and immunological detection techniques have been tried for shortening the detection time of pathogens [63,64]. However, the high cost, low sensitivity and cumbersome preparation process have always restricted their further use.

The SERS technology can be used to discriminate the pathogenic microorganism species at a higher colony level. In order to distinguish and identify the specific bacteria species at a lower concentration level, Raman scatting technology combined with statistical method and molecular recognition strategy have been developed [17,26]. Aptamers are a class of recognition molecules with specific selectivity that can improve the discrimination of the microorganism species. Researchers have successfully detected *Vibrio parahaemolyticus*, *Pseudomonas aeruginosa*, and *Salmonella typhimurium* by SERS technology combined with aptamers. 

**Figure 5:**
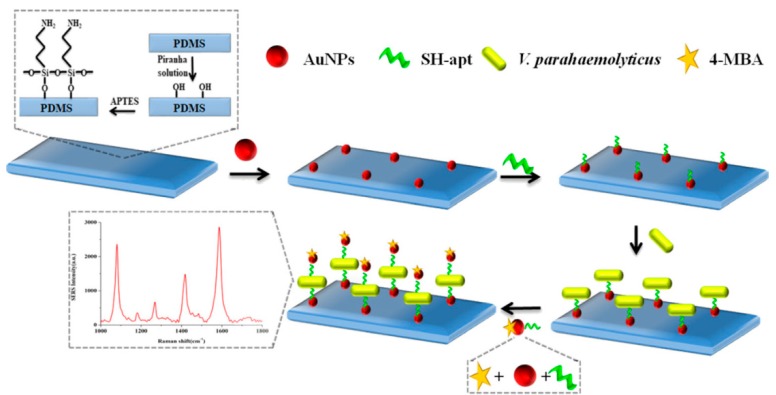
Schematic of SERS aptasensor based on Apt-Au-PDMS composites film for *Vibrio parahaemolyticus* determination. (Reprinted with permission from Springer, 2018 [65]).

Shen et al. developed a new detection method for *Vibrio parahaemolyticus* by sandwich method based on SERS analysis and aptamers [65]. The Au NPs coated polydimethylsiloxane film (Au-PDMS) was firstly prepared as SERS substrate. Aptamers responding to *Vibrio parahaemolyticus* were immobilized on the functionalized PDMS film by hybridization with the Au NPs through Au-thiol binding. The target *Vibrio parahaemolyticus* were then adsorbed onto the capture aptamers via the affinity. Additionally, the Au NPs modified with the aptamers and Raman detection molecules 4-mercaptobenzoic acid (4-MBA) were introduced by forming sandwich complexes of SERS aptasensor-target-4-MBA. The concentration of *Vibrio parahaemolyticus* showed a good linear relationship with the peak intensity of 4-MBA in the range of 1.2 × 10^2^ to 1.2 × 10^6^ CFU·mL^−1^ (Figure 5).

The sandwich technique was simple to operate and has been widely used in immunological assays. However, the detection sensitivity of this method has a strong relationship with the size of the target and the capture probe [66]. If the size difference between the capture probe and the target is too large, the contents of the target and the reporter probes adsorbed onto the capture probe would be unstable, and the detection sensitivity could not be maintained at a constant level. Therefore, the sandwich technique was not suitable for the detection of the large-scale target when using short-strand aptamer as capture probes. In this context, it was not difficult to understand that *Pseudomonas aeruginosa* ((0.5 – 1) × (1.5 – 4) μm) and *Salmonella typhimurium* ((0.6 – 0.9) × (1 – 3) μm) were detected by SERS and aptamers using the competition technique, rather than the sandwich technique.

**Figure 6:**
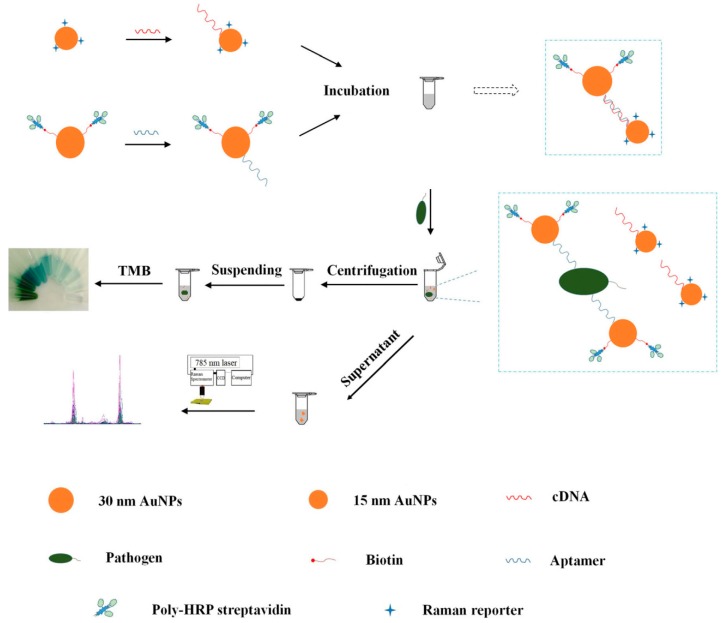
Schematic illustration of the fabrication procedure of the dual mode aptasensor for sensitive detection of *Pseudomonas aeruginosa*. (Reprinted with permission from Springer, 2018 [67]).

Wu et al. constructed a SERS/colorimetric dual mode aptasensor for the determination of *Pseudomonas aeruginosa* [67]. Two kinds of Au NPs with different sizes were prepared and used to modify the aptamer against *Pseudomonas aeruginosa* and its corresponding complementary DNA (cDNA). In the absence of *Pseudomonas aeruginosa*, the aptamer linked to the large scale Au NPs was hybridized with the cDNA linked to the small scale Au NPs. When the aptasensor was exposed to *Pseudomonas aeruginosa*, the aptamers dissociated from the cDNA due to the stronger interaction between the aptamer and *Pseudomonas aeruginosa*. Owing to the decreased electromagnetic effect, the larger sized Au NPs-aptamer complexes could be separated from the small sized Au NPs-cDNA complexes modified with Raman reporter after centrifugation. Therefore, the SERS signal from the supernatant decreased, while a new absorption peak at 640 nm can be determined after addition of hydrogen peroxide and 3,3′,5,5’-tetramethylbenzidine (TMB). The reliability of this approach was validated simultaneously by detecting different concentration gradient of *Pseudomonas aeruginosa* using Raman and UV-vis spectrophotometry. Besides, the approach showed a satisfying recovery result in the detection of spiked tap water and chicken meat samples (Figure 6).

**Figure 7:**
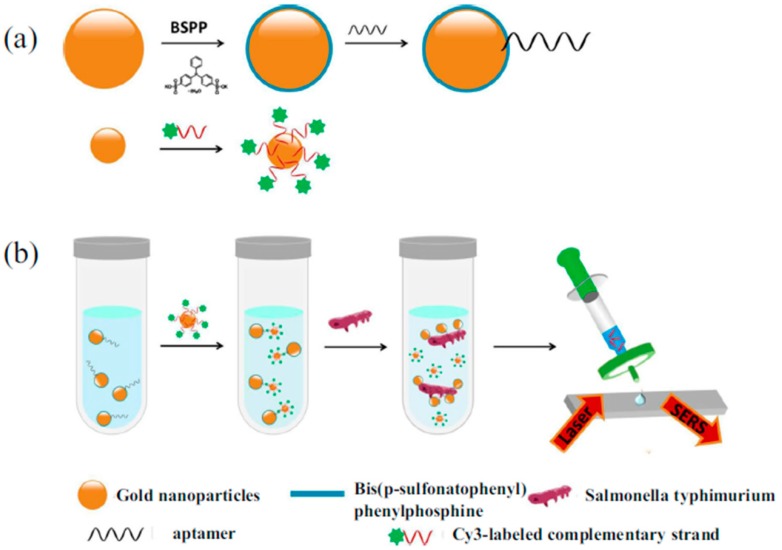
Schematic diagram of Au NPs based SERS aptasensors for the detection of *Salmonella typhimurium*. (**a**) Schematic diagram of the fabrication process of SERS aptasensors; (**b**) Schematic diagram of the detection process of *Salmonella typhimurium*. (Reprinted with permission from Springer, 2018 [68]).

Similarly, Xu et al. developed a rapid determination method for *Salmonella typhimurium* by use of competition technique [68]. In the absence of *Salmonella typhimurium*, the Raman signal intensity exhibited a significant enhancement because of the narrow gap between the aptamer modified larger-scale Au NPs and the complementary-sequence-modified smaller Au NPs. When the aptasensor was exposed to *Salmonella typhimurium*, the Raman signal intensity dropped because of the wide distance caused by the dehybridization of aptamers from their complementary strand. An exact linear relationship can be observed between the logarithm of the number of *Salmonella typhimurium* colonies and the Raman signal intensity at 1203 cm^−1^. The LOD value was 35 CFU·mL^−1^, while the detection time was less than 1 h (Figure 7).

### 3.3. Determination of Mycotoxins, Microcystin and Pesticide Residues

Due to biological eutrophication and the widespread use of pesticides, these pollutants are gradually becoming a great threat to drinking water and the environment throughout the world. Besides, as secondary metabolites secreted by filamentous fungi, mycotoxins are widely found in food, feed and other agricultural products, which also have a wide range of toxic effects on humans and animals. Therefore, the development of trace detection methods for mycotoxins, microcystin and pesticide residues is an evident necessity to human health. A large amount of efforts have been devoted to the detection of these poisonous materials, such as ELISA, HPLC, GC/MS, fluorescence and electrochemistry [69,70,71]. These methods have high sensitivity and accuracy; however, expensive reagents, skilled personnel and cumbersome operating procedures have hindered their practical applications. Nowadays, rapid, economic and sensitive methods based on aptamer sensors and SERS technologies have been developed for the detection of these hazardous material. The following aspects were introduced for these applications. 

**Figure 8:**
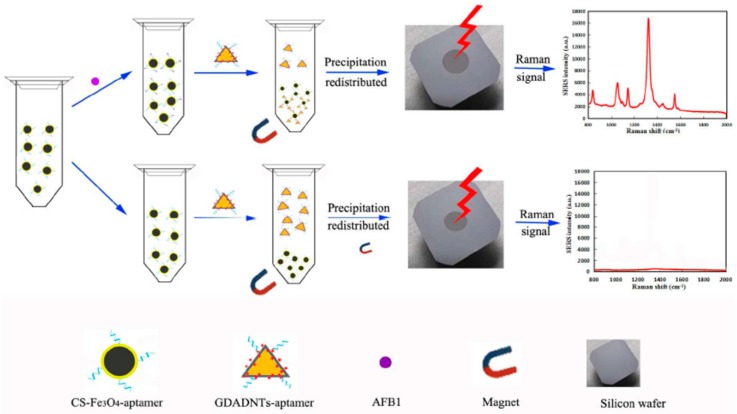
A universal SERS aptasensor platform for trace detection of AFB1. (Reprinted with permission from Elsevier, 2017 [72]).

The groups of Yang and Chen developed a SERS aptasensor based on Ag NPs core-shell nanotriangle (GNTs)/Ag) and chitosan modified Fe_3_O_4_ (CS-Fe_3_O_4_) magnetic-bead for trace detection of Aflatoxin B1 (AFB1) [72]. As shown in Figure 8, firstly, the AFB1 aptamers were coated onto the CS-Fe_3_O_4_ by using glutaraldehyde coupling, and the obtained CS-Fe_3_O_4_-aptamer was used as the capture nanoprobe and enrichment SERS-active substrate. Secondly, the core-shell GDADNTs were obtained by assembling the DTNB Raman reporter molecules onto the (GNTs)/agnanotriangles, and then the GDADNTs-aptamer reporter nanoprobes were formed by conjugating amino-terminal aptamers with the GDADNTs. In the presence of AFB1, the sandwich GDADNTs-aptamer-AFB1-aptamer-CS-Fe_3_O_4_ could be magnetically separated from the suspension and an evident SERS signal could be recorded. When there was no AFB1, the Raman signal could not be detected because the sandwich structures disappeared. Based on this principle, the intensity of Raman signal showed a good linear relationship with the concentration of AFB1 in the range of 0.001 to 10 ng/mL with the LOD as low as 0.54 pg/mL. Besides, the developed SERS aptasensor showed a good selectivity and a high stability with the RSD of ca. 5%. Apparently, the sensitivity and stability were favorable with other analogous biological labeling methods.

**Figure 9:**
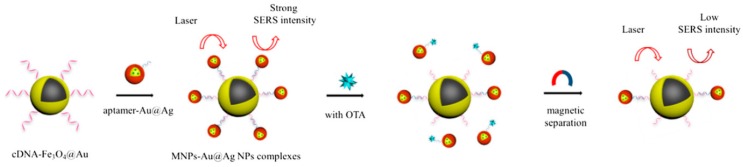
Schematic of the detection principle of the SERS-based aptasensor for ochratoxin A (OTA) detection. (Reprinted with permission from Springer, 2018 [73]).

The groups of Zhu and Long developed another SERS aptasensor for trace detection of ochratoxin A (OTA) by combining Fe_3_O_4_@Au NPs and Au-DTNB@Ag nanoprobes [73]. The OTA aptamer and the Raman signal probes DNTB were modified with Ag NPs, and the obtained aptamer-Au-DNTB@Ag NPs coupled with the cDNA-Fe_3_O_4_@Au composites by hybridization. Due to the hotspots between these two nanoparticle composites, a high Raman signal was recorded. While in the presence of OTA, the aptamer dissociated from the cDNA of the cDNA-Fe_3_O_4_@Au composites. After magnetic separation, the Raman signal intensity of precipitation decreased sharply. The decreased Raman signal intensity vs. the OTA concentration in the range of 1.20 pg·mL^−1^ to 3.31 μg·mL^−1^ displayed a linear relationship with a LOD of 0.48 pg·mL^−1^ (Figure 9). Similarly, Wang’s group also proposed a rapid, sensitive and economic method for the OTA detection by Au@Au-Ag composites and Fe_3_O_4_ magnetic NPs [74].

**Figure 10:**
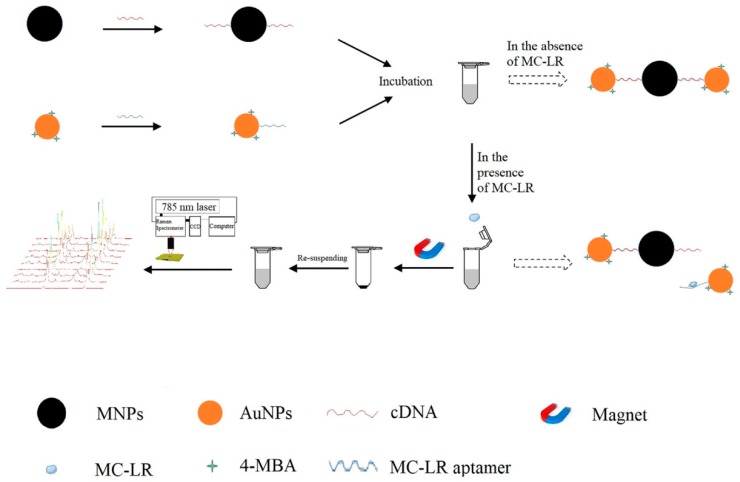
The principle of the analysis of MC-LR based on the SERS-based aptasensor. (Reprinted with permission from Elsevier, 2019 [75]).

The above literature show that the trace determination of mycotoxins can be attributed to the multiple Raman signal enhancement and the effective selectivity of the aptamer. The same determination strategy can also be found in the microcystin detection. Wu et al. used aptamer modified Au NPs and its cDNA modified MNPs for the detection of Microcystin-LR (MC-LR) by using Raman technology. In the absence of MC-LR, the Raman signal intensity was high because of the hotspots assembled by the hybridization of aptamer-Au NPs and cDNA-MNPs. In the presence of MC-LR, the signal intensity decreased because of the disappearing hotspots as a result of the dissociation of aptamer from its cDNA. Finally, the MC-LR concentration was determined by the decreased SERS signal intensity. Additionally, the proposed method was validated by detecting different levels of MC-LR spiked in tap water samples. The results from the method were close to those of the ELISA method because the *p*-value (*p* > 0.05) exhibited no significant difference [75] (Figure 10).

**Figure 11:**
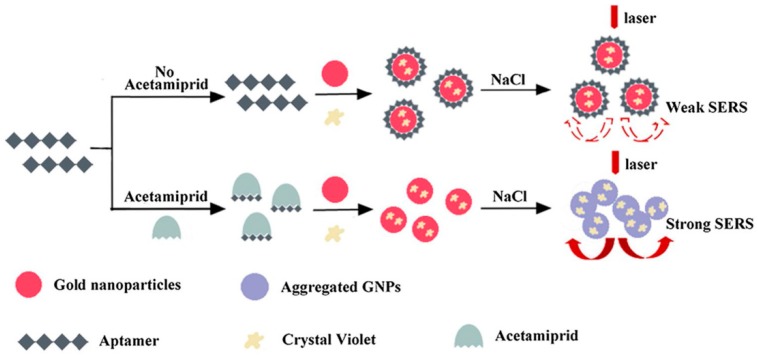
Schematic representation of the SERS-based biosensor for the detection of acetamiprid (AC). (Reprinted with permission from Springer, 2018 [76]).

Acetamiprid (AC), a kind of neonicotinoid insecticide, has been extensively used in agriculture fields. Extensive usage of AC can generate a potential risk of environments and human beings. Chen et al. skillfully utilized the specific recognition of aptamers for AC and the determination principle was based on the fact that naked Au NPs can be aggregated easily in a high-concentration salt solution [76]. In the absence of AC, crystal violet and aptamer would be attracted onto the naked Au NPs by electrostatic adsorption and a stronger coordination bond, respectively. Due to the protective effect of the aptamer, the modified Au NPs could not be aggregated closely in high-salt solutions. The solution displayed a purple color and the SERS intensity was weak. However, in the presence of AC, the specific binding of AC aptamer led to the dissociation of aptamer from the Au NPs. Losing the protection of the aptamer, the bare Au NPs would aggregate again in high-salt solutions, and an intense SERS signal could be determined. Undoubtedly, the increased SERS intensity was linear with the concentration of AC in the range of 3.0 × 10^−8^ to 4.0 × 10^−6^ M with an LOD of 1.76 × 10^−8^ M. Compared with the complicated covalent-modified Au NPs, the proposed method utilized naked Au NPs, which simplified the experimental operation and shorted the detection time. In addition, the variation in Raman signals caused by the aggregation or dissociation of NPs was also extremely sensitive. Therefore, the colorimetric detection of AC based on the SERS biosensor was facile and sensitive, which was successfully verified in different adulterated tea samples (Figure 11).

### 3.4. Determination of Antibiotics, Illicit Drugs, Hormones

The abuse of antibiotics, illicit drugs and hormones has been a worldwide problem that brings about severe societal consequences, such as reduced human immunity, increased treatment costs and damaged social stability. Studies have shown that drug abuse can affect the ultimate uptake of humans. The excessive accumulation in the body may result in varying levels of harm to human beings, such as nephrotoxicity, hepatotoxicity, allergic reactions, damage to the nervous system, high blood pressure, fetal teratogens, breast cancer risk, and so on [77,78]. Therefore, it is urgently necessary to introduce rapid, ultrasensitive and cost-efficient methods for the detection of antibiotics, illicit drugs and hormones. HPLC, GC/MS, surface plasmon resonance and ELISA methods have been used for the determination of these substances. While these methods are almost laboratory-based, the high costs of equipment, reagents and labor imposes limits on their practical application. Biosensors based on aptemers exhibit excellent performance in cost, sensitivity and detection speed, especially when combined with SERS technology. 

**Figure 12:**
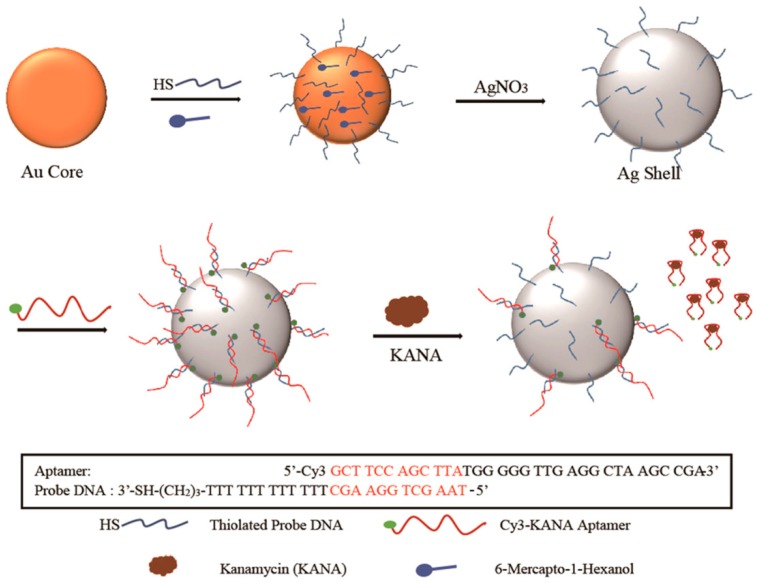
Schematic diagram of the SERS-based method for kanamycin (KANA) detection by using double-strand DNA aptamer-bonding Au@Ag NP. (Reprinted with permission from Elsevier, 2019 [79]).

Sun et al. introduced an aptamer with a terminal modified Raman probe Cyanine-3 (Cy3) for the detection of kanamycin (KANA) innovatively (Figure 12). The modified aptamers were immobilized onto the Au@Ag nanocomposites by hybridization, and the Raman intensity was high because of the core-shell structure of Au@Ag NPs. Due to the specific selectivity of aptamer, the Raman intensity decreased with the increment of the content of KANA in artificially spiked milk. This promising method could be used in trace determination of KANA residue with an ultra-low LOD of 0.90 pg/mL and a broad linear relationship in the range from 100 ng/mL to 10 μg/mL [79].

**Figure 13:**
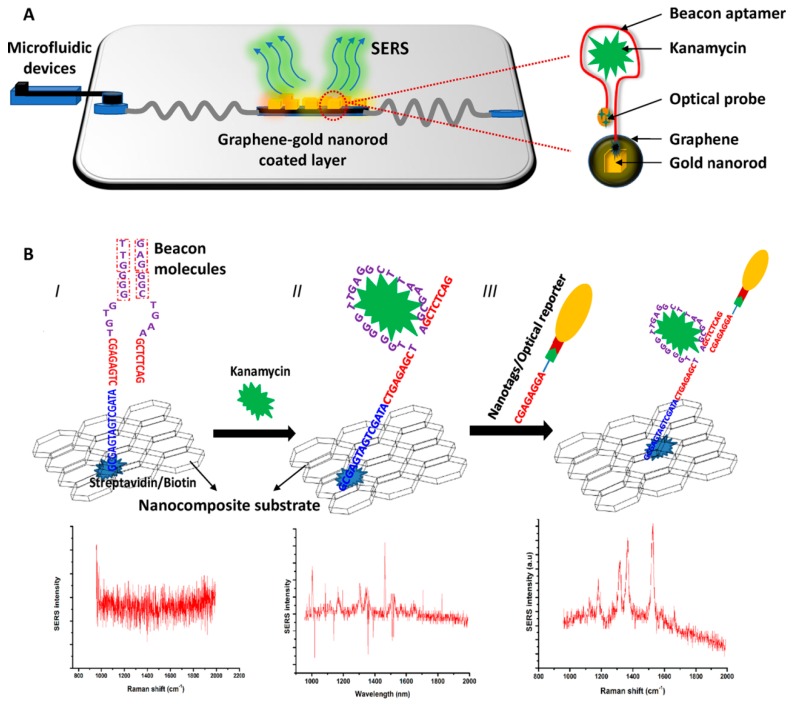
Scheme of the experimental platform for KANA detection. (**A**) Scheme of the experimental platform; (**B**) Illustration of the principle of the indirect assay. (Reprinted with permission from Elsevier, 2019 [44]).

Another aptamer-based SERS sensor detected KANA with the aid of GO for reducing the blinking effects effectively in Raman scattering [44]. The scheme for KANA detection by these nanocomposites is shown in Figure 13. In step I, the biotinylated aptamers for KANA are immobilized onto GO/Au-NPs nanocomposite by conjugation with streptavidin. In step II, the KANA is absorbed onto the GO/Au-NPs layer by the specific binding of the aptamer. In step III, the optical probes are immobilized onto the hotspots by hybridization. A microfluidic device was used for the detection of KANA at a small residue in tape water, drinking water, milk and orange juice. The results indicate that the Raman signal intensity had a good linear relationship with the increment of KANA concentration. Besides, the average recovery results calculated by the ELISA assay were similar with the requirements in food samples. Their research showed that the aptamer-based SERS sensors could be used for the detection antibiotics, and the blinking effect could be effectively reduced by rational use of nanomaterials.

**Figure 14:**
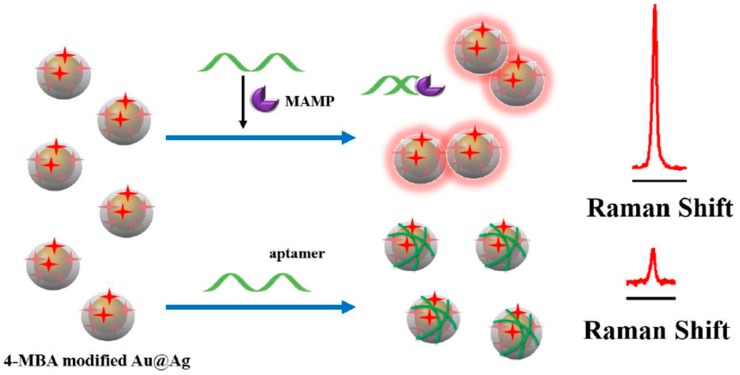
Schematic representation of SERS detection of methylamphetamine (MAMP) based on Au@Ag core-shell NPs. (Reprinted with permission from Elsevier, 2018 [80]).

Methylamphetamine (MAMP) is the second most widely abused illicit drug in the world, which exists in blood and urine matrices. Mao and co-workers [80] described a biosensor based on labeled Au@Ag NPs for detection of MAMP with SERS technology, and the results were compared with those from mass spectrometry. The detection principle is shown in Figure 14. The MAMP aptamers were analogously immobilized onto the labeled Au@Ag NPs based on the electrostatic interaction, making the modified Au@Ag NPs displace from each other and subsequently leading to a decrement of SERS intensity. In the presence of MAMP, the specific binding between the aptamers and MAMP kept the aptamers away from the labeled Au@Ag NPs. Consequently, the hotspots formed by the Au@Ag NPs effectively improved the SERS intensity, and the increased intensity showed a good linear relationship with the concentration of MAMP in the range from 0.5 ppb to 40 ppb. Importantly, the determination results in human urine sample by this new proposed method matched well with the same range of those from mass spectrometry. Their research indicated that the biosensor analysis can be potentially used in the rapid detection of MAMP in real samples. In addition, the aptamer-based SERS sensors can also achieved satisfactory test results in other drug detections [81].

**Figure 15:**
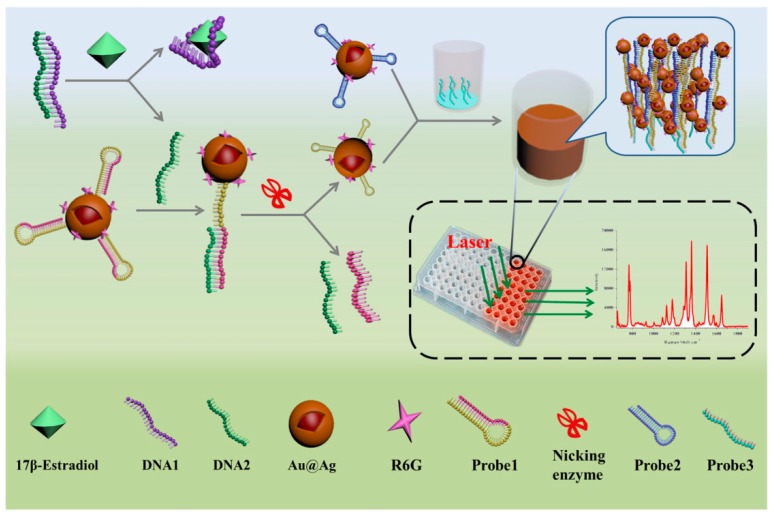
Schematic representation of aptamer-based SERS sensor for detection of 17 β-estradiol. (Reprinted with permission from Springer, 2019 [28]).

**Figure 16:**
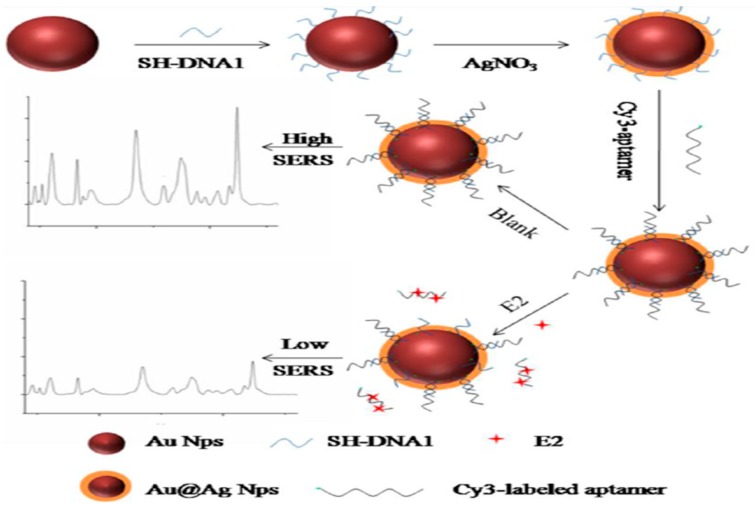
Schematic illustration for the E2 detection using SERS (E2 refers to 17 β-estradiol). (Reprinted with permission from Elsevier, 2018 [85]).

17 β-estradiol is a kind of common estrogen that has potential effects on normal physiological processes of human beings and animals, such as sexual developmental, pregnancy, cognitive behavior, etc. [82]. Traditional methods including HPLC-MS, GC-MS and ELISA have been used for the detection of 17 β-estradiol [83,84]. However, there are few articles on the aptamer-based SERS sensor for 17 β-estradiol detection. Chen et al. described an aptamer-based SERS sensor for the detection of 17 β-estradiol by hybridization chain reaction (HCR) [28]. As shown in Figure 15, probe 1 could automatically form a hairpin structure onto the labeled Au@Ag nanocomposite at first. In the presence of 17 β-estradiol, the single-stranded DNA1 preferably combined with 17 β-estradiol, making the DNA2 strand hybridize with the unhybridized probe 1. Subsequently, the partial dsDNA structure was hydrolyzed in the presence of nicking enzyme. Then the remaining probe 1 automatically formed a small hairpin again with a shorter stem. Finally, an HCR could occur after the addition of probe 3 and probe 2 in the ELISA plate, and a strong Raman signal could be detected in the wells. Conversely, it was not difficult to understand that the Raman intensity would not rise if there was no 17 β-estradiol. Under the optimal experimental conditions, the SERS signal at 1651 cm^−1^ increased linearly with the concentration of 17 β-estradiol in the range from 1 pM to 10 nM with an LOD as low as 0.1 pM. The developed method could be used for sensitive detection of 17 β-estradiol, but the cumbersome detection steps restricted its further use. In contrast, the approach of Pu and his coworkers was simpler [85]. The detection strategy was based on the hybridization on the Au@Ag nanocomposite (Figure 16). In the absence of 17 β-estradiol, the Raman signal was higher because the hybridization reaction between the Cy3-labeled aptamer and its complementary strand increased the content of the SERS probes on the composites. Conversely, the Raman intensity was decreased as the result of the specific selectivity of aptamer to 17 β-estradiol, and the content of 17 β-estradiol could be deduced from the decreased Raman intensity. It was gratifying that the proposed method was highly sensitive and selective for 17 β-estradiol. The LOD value was 2.75 fM, which was lower than that of Chen et al., and the detection steps were also greatly simplified.

### 3.5. Tumor Detection and Photothermal Therapy

**Figure 17:**
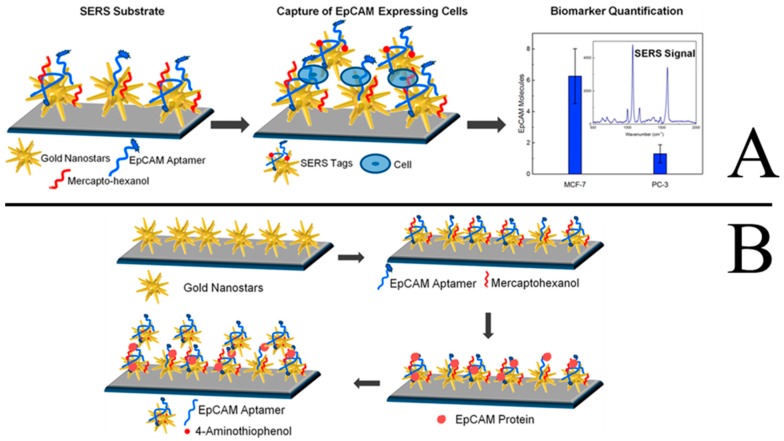
Schematic representation of the sequence of steps followed to carry out the EpCAM cells and protein assay (**A** and **B**, respectively). (Modified and reprinted with permission from ACS, 2018 [86]).

Cancer is almost the number one killer of people across the world, seriously threatening human health and life. The important strategies to improve the survival rate of most cancer patients are early diagnosis and effective therapies. A variety of cancer biomarkers, such as nucleic acids, proteins, epithelial cell adhesion molecules (EpCAM), as well as tumor cells, offer valuable insight into cancer progression. Therefore, various technologies, especially nanotechnology-based approaches, have been used for the detection of these markers and tumor cells in order to provide the disease-specific treatments as soon as possible. Recently, aptamer-based SERS sensors have been used for the early diagnosis of cancer with high sensitivity and selectivity. Bhamidipati et al. described a new aptasenor based on SERS technology for the detection the EpCAM proteins and cells with a high sensitivity [86]. The detection principles are shown in Figure 17A and 17B. Firstly, the Au nanostars are adsorbed onto the silanized glass slides. After backfilling the substrate with mercaptohexanol (MCH), the thiolated aptamers for EpCAM are immobilized onto the Au nanostars by Au-thiol binding. Thirdly, the as-prepared substrates are incubated and interacted with varying concentrations of EpCAM proteins and cells. Finally, the SERS signal can be determined after the addition of the Au nanostars are modified with EpCAM aptamers and SERS tags. By using these approaches, the lowest concentration of EpCAM protein could be detected at 10 pM, and the EpCAM cells could be detected at the single-cell level. The described substrate-based biosensor would improve the ability to monitor cancer progression in cancer cells, blood, and other body fluids, thus improving the ability for early diagnosis of cancers.

**Figure 18:**
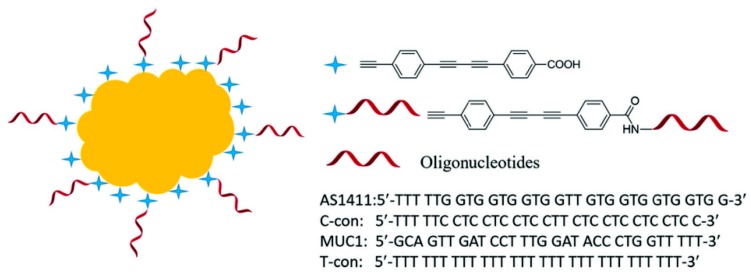
Schematic structure of the modified SERS nanoprobes in biologically Raman signalsilent region. Four oligonucleotides (AS1411, C-con, MUC1, and T-con) were anchored on Au NPs surface. (Reprinted with permission from Wiley, 2018 [87]).

Tang and his coworkers demonstrated another aptamer-based SERS nanoprobe for in vivo tumor cell spectral detection and imaging [87]. As shown in Figure 18, the oligonucleotide aptamers were conjugated with the SERS reporter at first, and the SERS reporter modified with oligonucleotide aptamers were then attached on the Au-NPs’ surface by C–Au binding. To illustrate the selectivity, another two oligonucleotides, C-con, and T-con were also anchored onto the Au-NPs’ surface. Raman imaging and bright view imaging results showed that the aptamer-based SERS nanoprobe could be selectively up-taken by the targeting cancer cells (MCF-7 cells) due to the specific interaction of aptamers and nucleolin protein on the MCF-7 cells’ surface, and the SERS intensity increased with the increased number of MCF-7 cells. Conversely, C-con and T-con oligonucleotides could not be uptaken by the MCF-7 cells because of the weak interaction; thus, the SERS intensity could not be observed. Therefore, the number of tumor cells can be detected according to the SERS peak at 2205 cm^−1^, and the LOD reached up to five cancer cells. This method for measuring the number of tumors at a low level could be potentially used in the early treatment of cancer.

**Figure 19:**
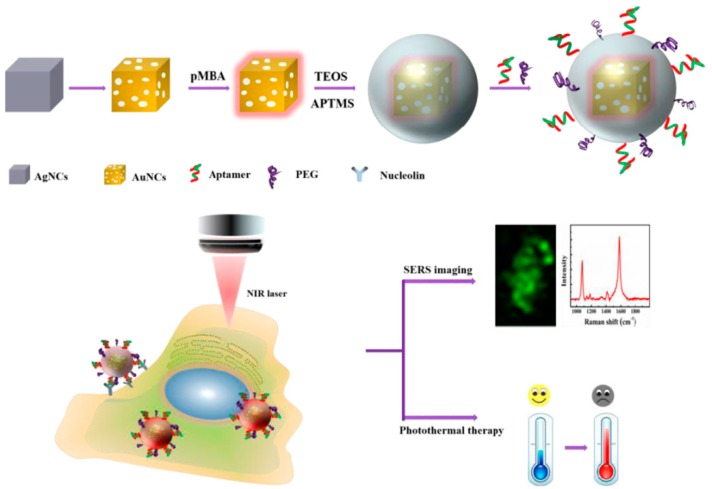
Schematic diagram of cellular SERS imaging and photothermal therapy. (Reprinted with permission from ACS, 2019 [91]).

Besides early diagnosis, the aptamer-based SERS sensors can also be used in the photothermal therapy of cancers. Photothermal therapy is a kind of method that gathers materials with high photothermal conversion efficiency around the tumor and converts light energy into heat energy to kill cancer cells. Compared with chemotherapy and radiotherapy, photothermal therapy had its unique advantages, such as low cost, site-specific controllability, high safety and remarkable curative effect [88,89,90]. The groups of Wu and Zhu developed an aptamer-conjugated Au nanocage (AuNC) for cellular SERS imaging and near-infrared photothermal therapy. As shown in Figure 19, *p*-MBA was used as SERS probe and was grafted onto the AuNC surface via Au-thiol binding. The outer shell of SiO_2_ improved the stability of *p*-MBA-encoded AuNCs and provided the binding sites for tagging a nucleolin-specific aptamer AS1411. According to the proposed method, the prepared AuNCs/SiO_2_/aptamer nanoprobes could be targeted to the nucleolin-positive expressed cells (MCF-7 cells) because of the specific recognition of AS1411 aptamers for nucleolin on the cell membrane of MCF-7 cells. Collectively, the as-prepared nanoprobes could selectively recognize MCF-7 cancer cells by SERS imaging, and the tumor cells could be killed by these nanoprobes upon NIR laser irradiation at a relatively low power density [91].

**Figure 20:**
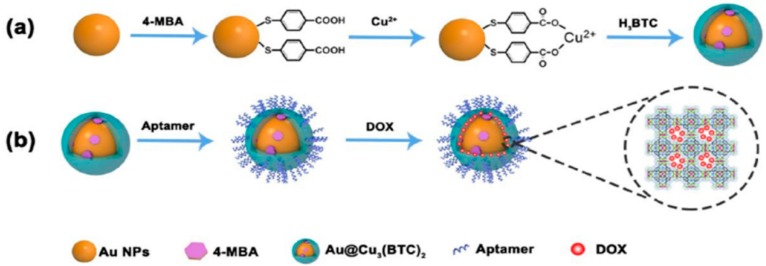
Diagram of preparation of Au@Cu_3_(BTC)_2_ NPs (**a**), and modification of Au@Cu_3_(BTC)_2_ NPs (**b**). (Reprinted with permission from RSC, 2019 [92]).

Li and Hu fabricated a kind of Raman probe modified nanoscale metal organic frameworks (NMOFs) and used it for Raman imaging and synergistic chemo-photothermal therapy [92]. Firstly, the Raman tag 4-MBA was assembled onto the Au NPs, and then the core-shell NMOFs were conjugated by linking of Cu_3_(BTC)_2_ and Au NPs. Secondly, the amino-modified aptamers were grafted onto the NMOFs by amidation reaction between the amino groups of aptamers and the carboxyl groups of NMOFs. Thirdly, the anticancer drug doxorubicin (DOX) were loaded with NMOFs and the Raman tag-bridged NMOFs were prepared. The as-prepared nanosensors showed an excellent SERS effect, high photothermal effect and drug loading capacity, which were successfully used in cell tracking and in vivo synergistic chemo-photothermal therapy of tumor, illustrating the possibility of early diagnosis and effective therapy of cancer (Figure 20).

Besides photothermal therapy, the aptamer-based SERS sensor was used in the photodynamic therapy of tumor. Liu et al. developed a novel photodynamic theranostic platform conjugated with DNA oligonucleotide for improving selectivity and sensitivity. By using this integrated nanoplatform, a remarkable enhancement in photodynamic therapy efficiency with low side effects can be observed. The as-prepared probe was successfully used in early monitoring and therapy of breast cancer [93]. In addition, folate was used as another specific recognition molecule for improving the photodynamic therapy efficiency of SERS sensor in tumor therapy [94]. Apart from this, there are some other therapy strategies for SERS sensors in photothermal ablation of bacteria [95], imaging and drug delivery [96]. These applications mean the SERS has been a powerful analytical technique in quantitative analysis and biotherapy in live cells and in vivo. It is not difficult to imagine that the SERS sensors based aptamer with good sensitivity and selectivity will certainly be more promising in the therapeutic field in the future.

## 4. Discussion and Conclusions

In this review, different synthesis methods of aptamer-based SERS sensors are discussed as well as their applications in the detection of small molecules, small ions, pathogenic microorganism, mycotoxins, microcystin, pesticide residues, antibiotic, illicit drugs, hormones, and tumor cells, together with photothermal therapy for tumor. Table 1 lists the detection applications of aptamer-based SERS sensors in this review. It is not difficult to observe that the enhancement of Raman signal is mainly based on the “hotspots” effect. In order to create hotspots, reasonable design and preparation of SERS substrates is extremely important. The paper has reviewed several kinds of common SERS substrates which can effectively improve the intensity of Raman signals. Besides, flexible chosen aptamers and regulation of salt concentration of the matrix can also create hotspots, effectively improving the sensitivity in Raman detection. However, excessive Raman activity may cause multiple interferences in detection. Therefore, improving the selectivity and specificity are common strategies in SERS detection applications. In the detection by using aptamer-based SERS sensors, the key criterion is high binding affinity of the aptamer to the target molecule. Since this binding affinity is often stronger than the interaction between the aptamer and its complementary strand, the competitive response based on these two effects is often applied during the detection. Additionally, some functional molecules, such as magnetic nanoparticles, graphene, etc., can effectively improve detection efficiency, which should be considered in practical applications. Apart from those points mentioned above, the aptamer-based SERS sensors can be effectively used in photothermal therapy of tumors because of the reduced side effects and high local therapeutic activity. 

## 5. The Challenges and Outlook of the Aptamer-Based SERS Sensors

The aptamer-based SERS sensors combine the high sensitivity of SERS with the high recognition ability of aptamer, which can effectively broaden the SERS detection application areas in drug quality control, environmental monitoring, pesticide detection, early diagnosis and treatment of tumors. However, the aptamer-based SERS sensors also have some shortcomings. For example, the types of aptamers for the target molecules are still relatively few, and some of their specificity is not satisfying, thus affecting the detection sensitivity and selectivity. Besides, the kinds of SERS substrates are also limited, and the fabrication method is a little complicated, which affects their universal usage. In addition, accuracy and stability are still major challenges for the targeted SERS sensors, especially in the mixed sample matrix application or at micro and trace detections. Therefore, the analysis of SERS sensors needs to make great progress in seeking more types of aptamers with good specificity, finding suitable Raman substrates with high stability, ease of operation and extensive expandability. In addition, the design and assembly of the aptamers with the SERS substrates is the key issue for the targeted SERS sensors. It is necessary to master the basic principles and design strategies in order to carry out promising sensors with low prices, strong enhanced effects and good repeatability. Moreover, improving detection accuracy and choosing the optimal pre-treatment process for eliminating the interference factors are necessary for using the aptamer-based SERS sensors in future. If this is the case, the detection and therapy application of the aptamer-based SERS sensors will be greatly expanded.

## Figures and Tables

**Table 1:** Applications of aptamer-based SERS sensors.

Target Analytes		Actual Samples	Linear Range	LOD	Ref.
Small molecules and ions	DEHP	tap water, bottled water, and a carbonate beverage	0.008–182 nM	8 pM	[60]
	Hg^2+^	laboratory water, rainwater, and pond water	0.25–10 nM	0.08 nM	[61]
	K^+^	rice samples	50–3000 nM	25 nM	[62]
Pathogenic microorganism	*Vibrio parahaemolyticus*	spiked prawn samples	1.2 × 10^2^ and 1.2 × 10^6^ CFU·mL^−1^	--	[65]
	*Pseudomonas aeruginosa*	spiked tap water and chicken meat	10^2^ and 10^6^ CFU·mL^−1^	20 CFU·mL^−1^ for SERS mode and 50 CFU·mL^−1^ for color mode	[67]
	*Salmonella typhimurium*	spiked milk	10^2^ to 10^7^ CFU·mL^−1^	35 CFU·mL^−1^	[68]
Mycotoxins, microcystin and pesticide residues	AFB1	peanut oil	0.001 to 10 ng/mL	0.54 pg/mL	[72]
	OTA	red wine and coffee	1.20 pg·mL^−1^ to 3.31 μg·mL^−1^	0.48 pg·mL^−1^	[73]
	MC-LR	tap water	0.01 to 200 ng/mL	0.002 ng/mL	[75]
	AC	green tea and adulterated tea	3.0 × 10^−8^ to 4.0 × 10^−6^ M	1.76 × 10^−8^ M	[76]
Antibiotics, illicit drugs, hormones	KANA	liquid whole milk	10 μg/mL to 100 ng/mL	0.90 pg/mL	[79]
	KANA	milk, orange juice, tape water, and drinking water	1 nM to 100 nM	0.75 nM	[44]
	MAMP	human urine sample	0.5 ppb to 40 ppb	0.16 ppb	[80]
	17 β-estradiol	human urine sample	1 pM to 10 nM	0.1 pM	[28]
	17 β-estradiol	aquaculture water, lake water and tap water	1.0 × 10^−13^ to 1.0 × 10^−9^ M	2.75 fM	[85]
Tumor	EpCAM	MCF-7 cells	500 nM to 10 pM for EpCAM protein, 10 and 5000 cells for MCF-7 cells	10 pM for EpCAM protein, the single cell level for MCF-7 cells	[86]
	MCF-7 cells	live mice	5–100 cell/mL	--	[87]

“--” refers to not mentioned in the original text.

## Abbreviations

SERS	surface-enhanced Raman scattering
NPs	nanoparticles
3D	three-dimensional
SELEX	systematic evolution of ligands by exponential enrichment
*p*-MBA	*p*-Mercapto benzoic acid
GO	graphene oxide
CNT	carbon nanotube
DEHP	bis(2-ethylhexyl)phthalate
ELISA	Enzyme linked immunosorbent assay
MNPs	magnetic particles
DTNB	5,5′-Dithiobis-(2-nitrobenzoic acid)
LOD	limit of detection
VB4r	Victoria blue 4R
AgNR	Ag nanorods
GLC	glucose
VBB	Victoria blue B
Au-PDMS	Au NPs coated polydimethylsiloxane film
4-MBA	4-mercaptobenzoic acid
cDNA	complementary DNA
TMB	3,3′,5,5′-tetramethylbenzidine
CS-Fe_3_O_4_	chitosan modified Fe_3_O_4_
AFB1	Aflatoxin B1
(GNTs)/Ag	Ag NPs core-shell nanotriangle
OTA	ochratoxin A
MC-LR	Microcystin-LR
AC	acetamiprid
HPLC	high performance liquid chromatography
GC/MS	gas chromatography-mass spectrometry
Cy3	Cyanine-3
KANA	kanamycin
MAMP	Methylamphetamine
HCR	hybridization chain reaction
EpCAM	epithelial cell adhesion molecule
MCH	mercaptohexanol
AuNCs	Au nanocage
NMOFs	nanoscale metal organic frameworks
DOX	doxorubicin
